# Effects of Postmortem Interval on Mouse Ovary Oocyte Survival and Maturation

**DOI:** 10.1371/journal.pone.0098384

**Published:** 2014-05-29

**Authors:** Guang-Li Zhang, Jun-Yu Ma, Quan Sun, Meng-Wen Hu, Xiu-yan Yang, Si-Hua Gao, Guang-Jian Jiang

**Affiliations:** 1 Diabetes research Center of Beijing University of Chinese Medicine, Beijing, China; 2 Department of Biochemistry and Molecular Biology, Hebei United University, Tangshan, China; 3 Department of Animal Science and Technology, Qingdao Agricultural University, Qingdao, China; 4 State Key Laboratory of Reproductive Biology, Institute of Zoology, Chinese Academy of Sciences, Beijing, China; Institute of Zoology, Chinese Academy of Sciences, China

## Abstract

To study the time- and temperature-dependent survival of ovarian oocytes collected from postmortem carcass, ICR mice were killed and placed for different periods (0, 1, 2, 4, 6, 8 and 10 h) at different temperatures (25°C, 4°C and 37°C). After preservation, oocyte morphology, germinal vesicle (GV) oocyte number, oocyte meiotic maturation percentage, mitochondrial distribution and intracellular glutathione (GSH) level were evaluated. The results showed no surviving oocytes could be collected by 2h, 6h, and 12 h after carcass preservation at 37°C, 25°C and 4°C, respectively. The number of collected GV oocytes in the ovary deceased as the preservation time lasted at the same temperature. Meanwhile at the same point in time, the ratio of germinal vesicle breakdown (GVBD) and the first polar body emission (PBE) gradually reduced as preservation temperature increased. In addition, the percentage of abnormal mitochondrial distribution in the preserved oocytes was obviously higher than that in the control oocytes, while GSH level was not altered in collected oocytes. Unexpectedly, neither chromosome arrangement nor spindle organization was affected as long as the oocytes from preserved carcasses could complete maturation. These data are helpful for proper use of ovary oocytes from postmortem carcass of valuable individuals.

## Introduction

Experimental animals play an important role in scientific research. Most of the oocytes used for studies are from slaughtered animals. Ovaries are usually removed immediately after the death of animals, but sometimes it is not convenient to conduct immediate experiments and oocyte may be taken from animals that die for a period of time. In addition, ovary oocytes of treasured animals including genetically modified individuals that accidentally die may need to be utilized to produce offspring. The length of time after the death of animals may be one of the important factors affecting oocyte quality. Schroeder et al. compared the capacity of oocytes matured *in vitro* following isolation at the GV stage from freshly killed mice with that of oocytes from the carcasses of mice killed more than 3 hours earlier. They found that maturation did not differ between oocytes from freshly killed mice and those collected from mice killed 6 hours earlier [1]. But Miao et al. showed that cell number and blastocyct percentages declined after *in vitro* maturation and fertilization when ovaries were removed at 30 minutes and 3 hours postmortem [2]. It is necessary to clarify the postmortem time effect on ovary oocyte vitality.

In addition, low temperature freezing is the common practical method for preservation of oocytes. It has been proved that oocytes are highly sensitive to the low temperature [3], and the formation of ice crystals is recognized as the main reason for the high sensitivity to low temperature [4]. Low temperature may also affect subsequent nuclear and cytoplasmic reorganization of the GV oocytes [5] and even damage mitochondria [6] and microtubules including those comprising the meiotic spindle [7]. These problems hamper the application of oocyte preservation. Thus it is necessary that research the effect of different temperature on oocyte vitality after the death of animals.

The objective of this study is to explore the effect of length of time of mouse carcass preservation after death at different temperatures on oocyte survival. We hypothesize that oocyte survival and quality are affected by carcass preservation time and temperature, and aim to find the postmortem time and temperature that can preserve the oocyte for in vitro maturation utilization. To assess the quality of preserved oocytes, oocyte number and morphology, glutathione content, and mitochondria after preservation and spindle configuration after culture were evaluated. Our study may provide the useful evidence for rescue of oocytes from deceased valuable or endangered mammals.

## Materials and Methods

All chemicals and culture media were purchased from Sigma Chemical Company (St. Louis, MO, USA) unless stated otherwise.

Mice were housed in 12-hour alternating light/dark cycles, with free access to water and food. All experiments were conducted with the approval of the Animal Research Committee of the Institute of Zoology, Chinese Academy of Sciences, China.

### 1. Oocytes collection and culture

ICR mice were killed by carbon dioxide euthanasia and carcasses were kept always at a controlled room temperature (25°C), low temperature (4°C) and high temperature (37°C) unless otherwise specified. Oocytes were collected, washed three times with M2 medium and cultured in M2 medium covered with liquid paraffin oil at 37°C in an atmosphere of 5% CO_2_. The oocytes were collected at different times for immunostaining or other detection.

### 2. Immunofluorescence

For spindle and chromosome analysis, oocytes were collected and fixed in 4% paraformaldehyde in PBS for 30 min and permeabilized in 0.5% Triton-X-100 at room temperature for 20 min. Then oocytes were blocked in 1% BSA supplemented PBS for 1 h and samples were incubated for 2 h at room temperature with fluorescein isothiocyanate-conjugated α-tubulin antibody (1∶300) to visualize microtubules in the spindle. After washing three times in PBS containing 1% Tween-20 and 0.01% Triton-X 100, the oocytes were stained with PI (10 µg/ml) for 10 min. Finally, oocytes were mounted on glass slides and viewed under a confocal laser scanning microscope (Zeiss LSM 710).

For mitochondrial staining or inner mitochondrial membrane potential staining, oocytes were cultured in M2 medium containing, 200 nM MitoTracker Red for 30 min at 37°C, 5% CO2 in air [8]. After staining was completed, the staining solution was replaced with fresh M2 medium, and oocytes were stained with Hoechst33342 (10 µg/ml) for 10 min. Finally, oocytes were mounted on glass slides and viewed under a confocal laser scanning microscope (Zeiss LSM 780).

### 3. Measurement GSH content

The intracellular content of GSH, the level of glutathione, was measured as described by Ge et al.[9]. After postmortem carcass preservation, GV oocytes were collected and washed three times in phosphate buffered solution (PBS). Thirty oocytes composed one sample and three samples were assayed for each treatment. Oocytes were added in 5 µl of 1.25 M phosphoric acid and vortexed thoroughly. Samples were then frozen at Liquid nitrogen and thawed at 37°C in a water bath. This procedure was repeated at least two times. Then the samples were placed in an ice bath for 5 minutes and centrifuged at 10,000 rpm for 10 minutes at 4°C. Absorbance was monitored continuously at 412 nm with a spectrophotometer for 25 min, with readings recorded every 5 min. Standards (1.0, 2.0, 5.0, 10.0, 25.0 and 100.0 µM) of GSH and a sample without GSH were also assayed. The amount of glutathione in each sample was divided by the number of oocytes [10].

### 4. Data analysis

For each treatment, at least three replicates were run. Statistical analyses were carried out by SPSS. Differences between treatment groups were evaluated with T-test. Data are expressed as mean SE and P<0.05 is considered significant.

## Results

### 1. Effects of different carcass preservation periods on oocyte meiotic process

**Figure 1:**
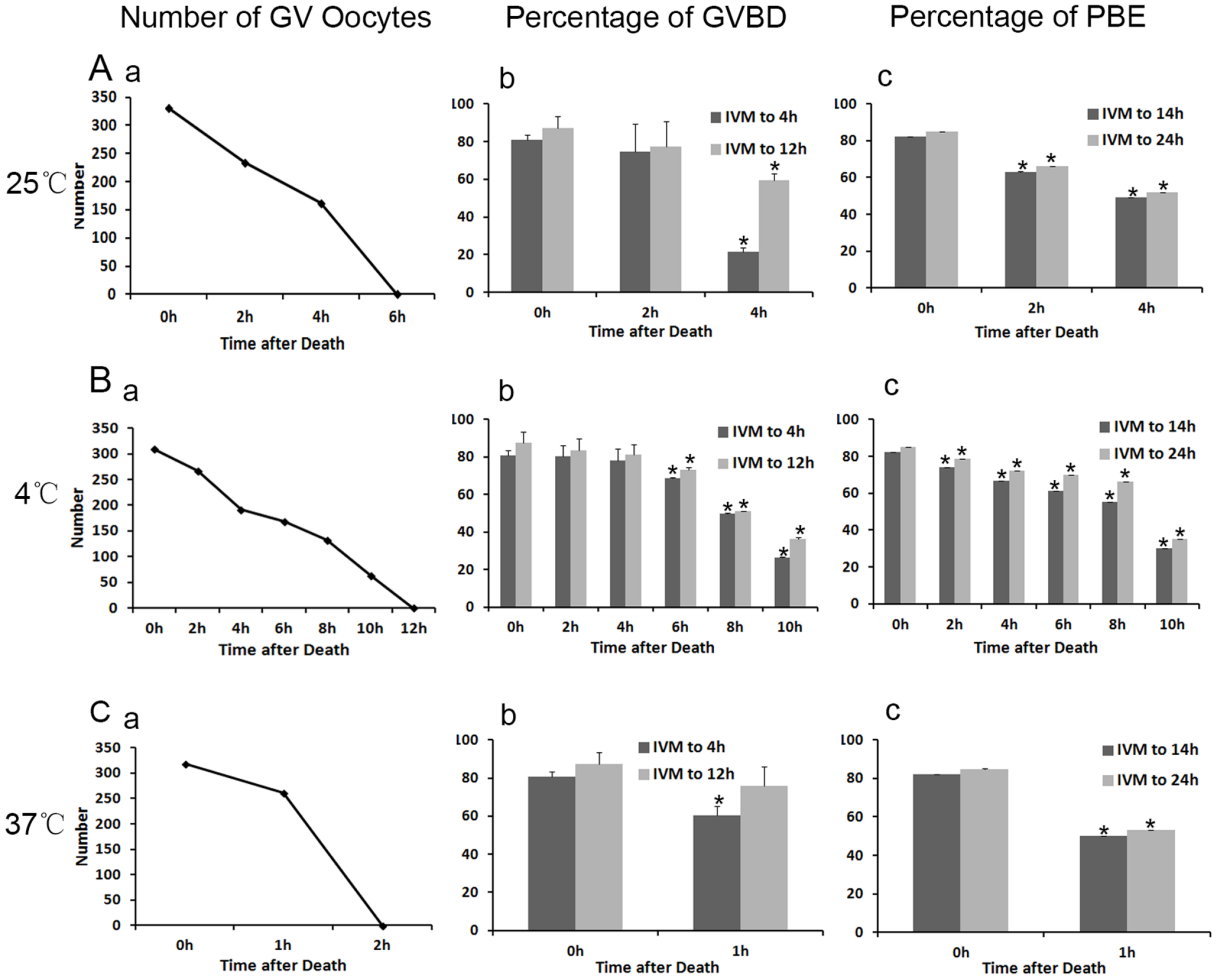
Effects of different carcass preservation periods on oocyte meiotic process. (A) oocytes matured in vitro after carcass preserved for different times (0, 2 and 4 h) at room temperature (25°C). (a) The number of GV oocytes collected from the preserved carcass in the experimental group and control group. (b) The percentage of GVBD oocytes in the control group (n = 330), PMI 2 h group (n = 234) and PMI 4 h group (n = 162). Each bar represents mean ±SEM (n = 3). * indicate statistically significant differences (P<0.05). (c) The percentage of PBE in oocytes cultured in vitro in preservation group and control group. * P<0.05. (B a) The number of GV oocytes collected from the ovary after preservation at low temperature (4°C). (B b) The percentage of GVBD oocytes in the control group (n = 309) and the preservation group (n = 267, 192, 168,132 and 63). * P<0.05. (B c) The percentage of PBE in oocytes cultured in vitro in different groups. * P<0.05. (C) The number of GV oocytes, percentage of GVBD oocytes and PBE same as A at high temperature (37°C). * P<0.05.

To study the oocyte meiotic maturation ablity, we counted the percentages of GVBD and polar body extrusion (PBE) which were the most obvious indicators of oocyte maturation. ICR mice were killed and carcasses were kept at a room temperature (25°C) for different hours, Fig. 1Aa showed that the number of collected GV oocytes decreased with preservation time. GV oocytes were not found in the field of vision when the carcass was kept for 6h or longer. When collected oocytes were cultured for 4 h, the percentage of GVBD oocytes in postmortem interval (PMI) 4 h group (21.42±2.11%) was significantly lower than that in the control group (80.76±2.71%, Fig. 1Ab). Then we cultured oocytes for 12 h. The number of GVBD oocytes similarly reduced in PMI 4 h group (59.29±3.89%) compared with that in the control group (87.33±6.22%, Fig. 1Ab). Next we examined the percentage of PBE after oocytes had been cultured for 14 and 24 h, on the basis of GVBD oocytes. We found that PBE was also remarkably reduced after carcass preservation (82.21±0.03% and 84.95±0.01% in the control group, 63.18±0.06% and 66.24±0.05% in PMI 2 h, 49.4±0.009% and 52.03±0.009% in PMI 4 h group, P<0.05,Fig. 1Ac).

### 2. Effects of different carcass preservation temperatures on oocyte meiotic process

Next we studied the ovarian oocyte survial after preservation of carcasses at 4°C. As shown in Figure 1B, the number of collected GV oocytes decreased with preservation time, and no oocytes could be collected in PMI 10 h group (Fig.1Ba). From 6 h after carcass preservation,,the percentage of oocyte GVBD was significantly decreased after oocytes were cultured for 4 h or extended 12 h (P<0.05). The oocyte maturation percentage was also decreased with the extension of carcass preservation time. Oocytes collected from postmortem carcass showed evidently decreased maturation percentage from 2 h preservation. (P<0.05, Fig. 1B c).

When ICR mice were killed and carcasses were kept at a high temperature (37°C) until no oocytes were found in the field of vision by 2 h. In the PMI 1 h group, the total number of GV oocytes was considerably lower than that in the control group (Fig.1C a). After cultured for 4 h, 80.76% of the control oocytes underwent GVBD, while 60.45% of the oocytes in the PMI 1h group did (Figure 1Cb, P<0.05). The oocyte maturation percentage was also significantly decreased. (P<0.05, Fig.1Cc).

**Figure 2:**
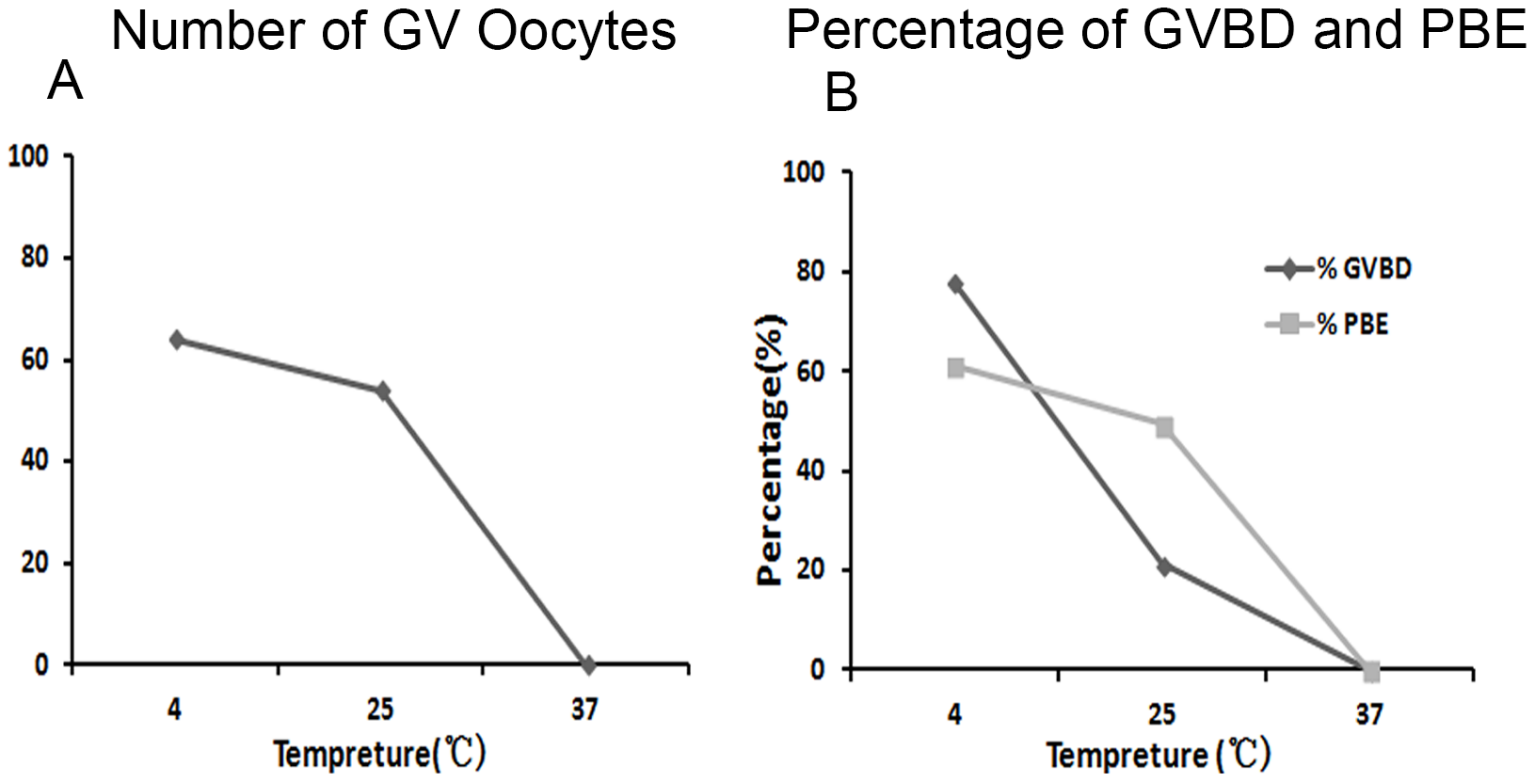
Effects of different carcass preservation temperatures on oocyte meiotic process. (A) The number of GV oocytes collected from mouse carcass after preserved for 4 hours at different temperatures. (B) The percentage of GVBD oocytes and PBE in PMI 4 h group at different preservation temperatures. Each bar represents mean ±SEM (n = 3). * P<0.05.

Figure 2 summarized the the number of oocytes collected from the ovary in PMI 4 h at different temperature. As shown in Figure 2A, the number of GV oocytes in low temperature group (n = 192) was maximal among three groups, while that in high temperature group was the minimal (n = 0). The ability of oocytes to undergo GVBD and maturation was also decreased with temperature increase (Fig. 2B).

### 3. Effects of different carcass preservation periods and temperature on spindle configuration in mouse oocytes after *in vitro* maturation (IVM)

**Figure 3:**
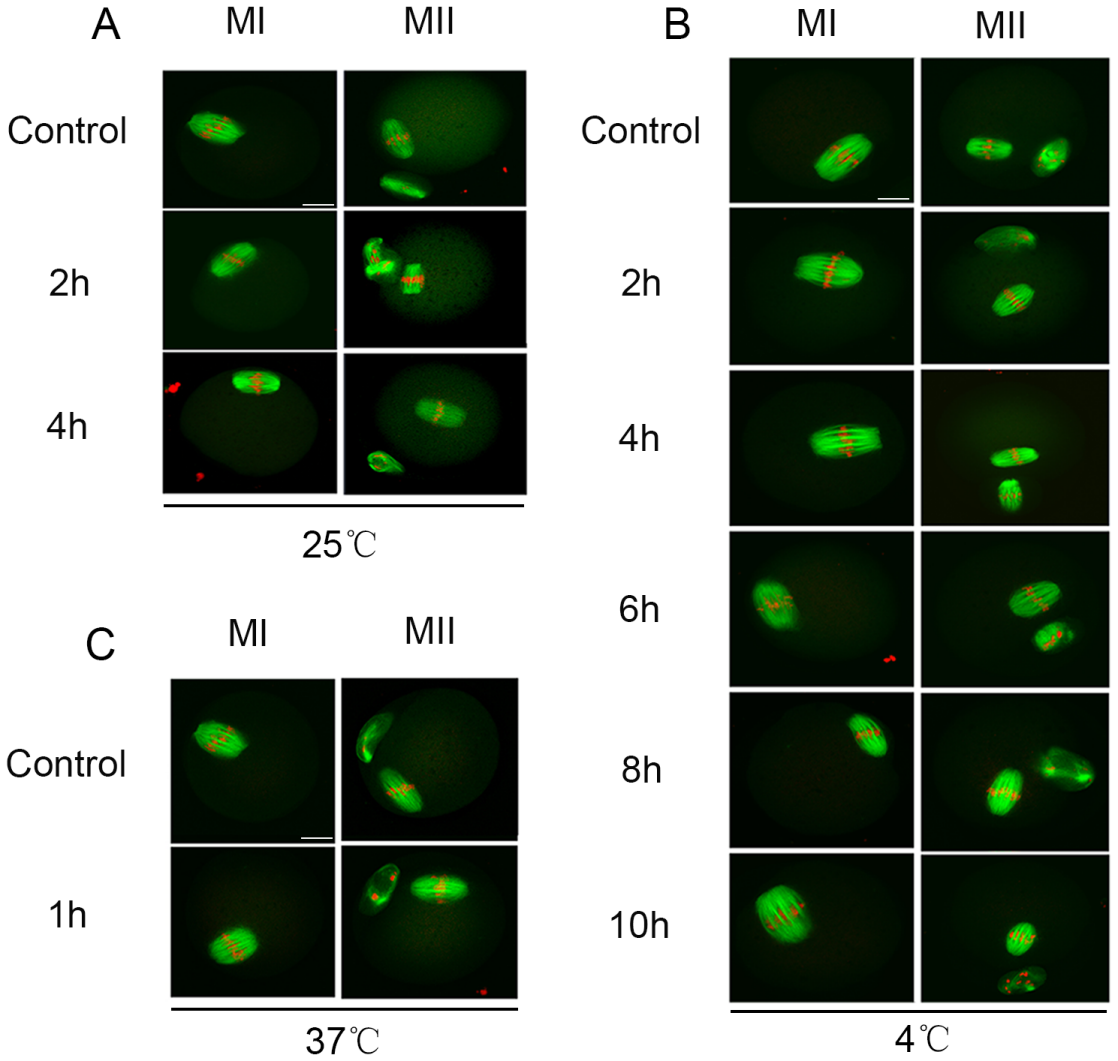
Effects of different preservation periods and temperatures on oocyte spindle configuration after IVM. Oocytes cultured in M2 medium for 8h (MI) and 12h (MII), spindle was stained with FITC-α-tubulin and DNA was stained with PI. (A, B,C) the normal spindle in mouse MI and MII oocyte in control group and experimental group at different preservation temperatures.

We examined the spindle and chromosomes of MI and MII oocytes from different preservation periods at different temperature by confocal scanning microscopy. Unexpectedly, we did not find abnormalities in spindle morphology and chromosome arrangement inoocytes from preserved carcasses. As shown in Figure 3, at a room temperature (25°C), most of the MI and MII oocytes exhibited fusiform spindles and well-aligned chromosomes, and there was no difference between the experimental group and the control group. Similarly, most of the MI and MII oocytes developed from the GV oocytes from carcasses preserved at low (4°C) or high (37°C) temperature also exhibited normal spindles and chromosomes.

### 4. Effects of different carcass preservation periods and temperatures on mitochondria distribution

**Figure 4:**
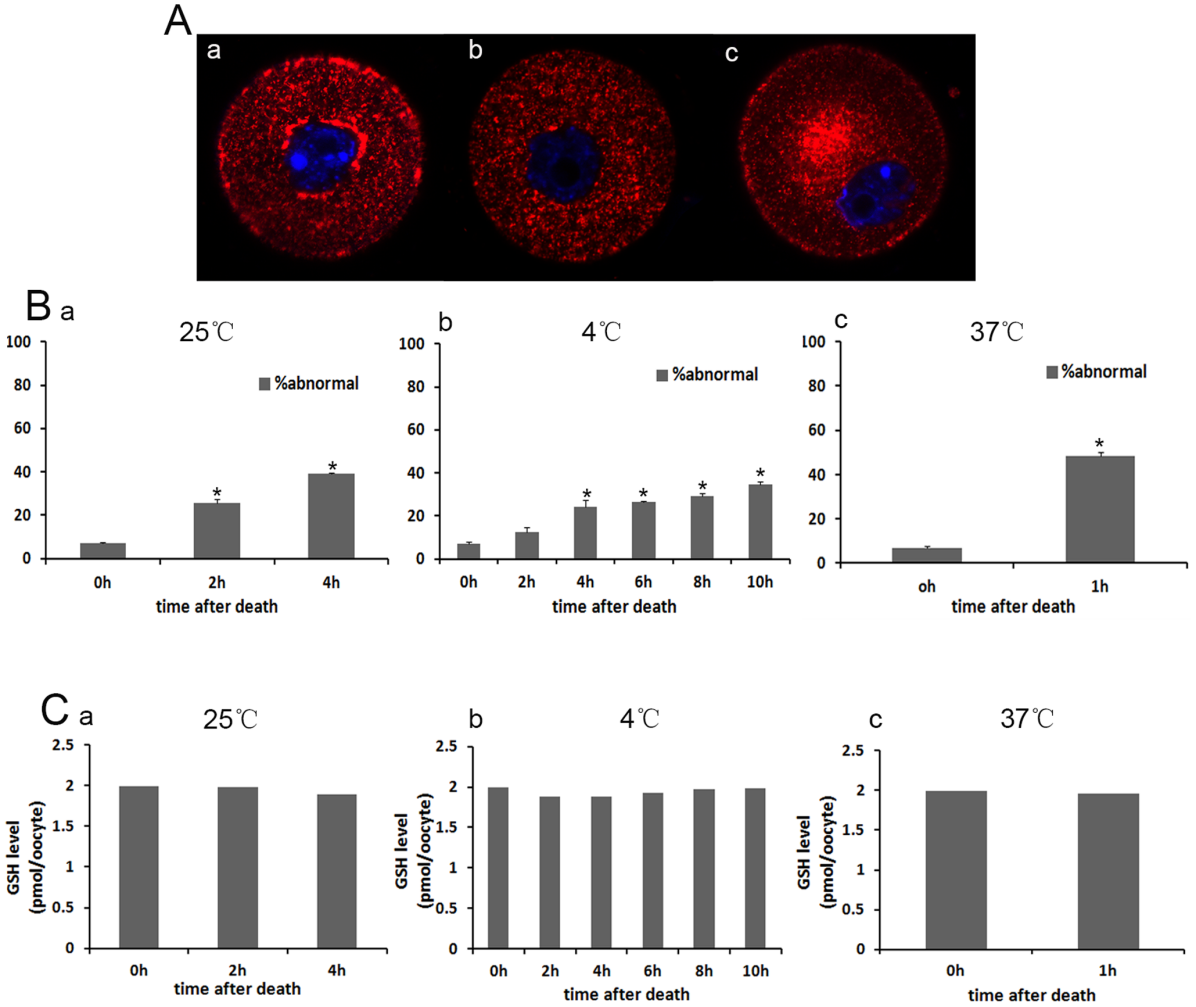
Effects of preservation time and temperature on mitochondrial distribution and GSH level in mouse oocytes. (A) Mitochondrial distribution was evaluated by fluorescence microscopy. Three localization patterns were observed: perinuclear, homogenous and clustered. (B) The percentage of abnormal mitochondrial distribution in GV oocytes in the control group (n = 279) and preservation group at different temperatures. * indicate statistically significant differences (P<0.05). (C) The GSH concentrations in GV oocytes (n = 30) in different groups.

We next examined the distributions of mitochondria in mouse oocytes using Mito-Tracker Red CMXRos staining. As reported previously [11], GV oocytes were categorized into one of three categories based on their mitochondrial distribution: Group 1, perinuclear and surrounding the GV (normal); Group 2, homogenous and throughout the entire ooplasm; and Group 3, clustered in the cytoplasm (abnormal) (Fig.4A). At room temperature in the control group (n = 279), only 6.86% of oocytes showed abnormal mitochondrial distribution. While in the preservation group (PMI 2 h and 4 h) (n = 261 and 267), 25.53% and 38.99% showed abnormal mitochondrial distribution (P<0.05, Fig. 4Ba). When carcass preserved at low temperature (4°C), 12.47% of oocytes showed abnormal mitochondrial distribution in PMI 2 h group (n = 270), 24.39% in PMI 4 h group (n = 249), 26.49% in PMI 6 h group (n = 198), 29.27% in PMI 8 h group (n = 144) and 34.61% in PMI 10 h group (n = 63), all higher than that in the control group (Fig. 4B b). There were significant differences between control group and preservation groups except PMI 2 h group (P<0.05). When carcasses were preserved at 37°C, about half (48.45%) of oocytes showed abnormal mitochondria distribution in PMI 1 h group (n = 219) (P<0.05, Fig. 4Bc).

### 5. Effects of different carcass preservation periods and temperatures on GSH level

We determined whether or not the antioxidant capacity of oocytes was impaired. When mouse carcasses were preserved for different times at different temperatures (25°C, 4°C and 37°C), the content of intracellular GSH was slightly lower than in control oocytes, but there was no statistical difference between each group (P>0.05, Fig. 4C).

## Discussion

The main aim of the present study is to determine the influence of carcass preservation time and temperature on oocyte survival and quality. We show that at the same temperature delayed excision of ovary not only decreases the number of surviving GV oocytes but also reduces the GVBD and maturation percentages after culture *in vitro*. We also find that low temperature preservation of carcass can extend oocyte survival time, while high temperature shows the opposite effect. The percentage of abnormal mitochondrial distribution in GV oocytes increases along with the extended preservation time at different temperatures. While GSH level was not affected. Unexpectedly, both spindle organization and chromosome configuration are normal in oocytes from preserved carcass as long as they can complete maturation in vitro.

Previous reports showed that mouse ovarian tissues stored *in vitro* before cryopreservation retained viable follicles up to 12 hours after death, whereas tissues left in situ had significantly reduced follicle numbers within 3 hours of death [12], [13]. It was also showed that GV oocytes chromatin configuration changed 5–10 hours after horse death, causing degradation of a large number of oocytes [14]. When mouse ovaries were excised at 30 min after death, percentages of atretic follicles increased while blastocyst cell number declined significantly after oocyte maturation in vitro [2]. We extended the carcass preservation time to determine how long the oocytes can keep survive at different temperatures. We found that no surviving oocytes could be collected by 2h, 6h, and 12 h after carcass preservation at 37°C, 25°C and 4°C, respectively. This indicates that oocytes in the ovary of deceased individuals lose viability sooner than in vitro. We speculate that the deceased body may release certain substances after death which accelerate the decomposition of oocytes in the ovary. At the same temperature, when oocytes collected at different time points were cultured *in vitro*, the percentages of GVBD and first polar body extrusion were both significantly decreased with the extended carcarss preservation. Meanwhile, oocytes maturation delayed in experimental group. The phenomenon shows that the extension of carcass preservation time can reduce the percentage of oocytes survival and maturation.

By comparing the different preservation temperature at the same time, our results showed that the percentage of GVBD oocytes in low temperature (4°C) group was higher than that in room temperature (25°C) group. And the percentage of GVBD oocytes in high temperature (37°C) group was the lowest among the three groups. Similarly, the percentage of the first polar body extrusion was the highest at low temperature (4°C), the lowest in high temperature (37°C) group. This indicates that low temperature can keep the vitality longer of ovarian oocytes after death of mouse.Our recent study showed that the survival percentage of porcine oocytes stored at 20°C was higher than at 37°C in TCM-199 with serum after 48 hours, and that 27.5°C was the optimal temperature for storing porcine oocytes in PFF or FCS [15]. Low temperature may reduce some pathological reaction of mouse body after death. Thus, low temperature is favorable to keep the vitality of mouse ovarian oocytes after death.

Next we determined the ovarian oocyte quality after carcass preservation Spindle appearance and GSH content are regarded as indicators of oocytes quality. Our results showed that the spindles of MI and MII oocytes were not affected in oocytes from preserved carcasses at different temperature compared with the control group. Cytoplasmic GSH content was also not altered. This is unexpectedly, since we believe that oocyte quality would be decreased in deceased ovary. The results indicate that spindles may be properly organized as long as the oocytes have the ability to complete maturation. Whether such oocytes can support embryo development needs further clarification. Expectedly, less than one-tenth of the fresh GV oocytes showed the abnormal mitochondria distribution, while about half of the preserved oocytes' mitochondria distribution was abnormal at high temperature (37°C). At the same temperature, the longer the preservation time, the higher the ratio of abnormal mitochondria distribution. The abnormal distribution of mitochondria may hint the decreased cytoplasmic quality of preserved oocytes. Dysfunction of mitochondria often appeared in bad oocytes.

In summary, our studies show that the mouse carcass can be preserved at room temperature (25°C) for 4 h without oocyte evident cytoplasmic damage. At a low temperature (4°C) the preservation time can be for 10 h, while carcass can only be preserved for 1 h at a high temperature (37°C). Low temperature carcass preservation can extend the mouse oocyte vitality, and the high temperature shows opposite effect. With the preservation time extension, the quantity and quality of GV oocyte are all reduced. Most of the preserved oocytes which retain the potential to mature *in vitro*,have normal spindle formation and GSH level, but the mitochondria distribution is abnormal, which may hinder the developmental competence. The findings may be useful for save of oocytes from deceased valuable or endangered animals.
